# Integrating Space Sexology Into Long-Duration Mission Architecture: A Five-Pillar Operational Framework

**DOI:** 10.7759/cureus.105103

**Published:** 2026-03-12

**Authors:** Piercarlo Minoretti

**Affiliations:** 1 Occupational Health, Studio Minoretti, Oggiono, ITA

**Keywords:** aerospace medicine, bioethics, crew preparedness, erobotics, habitat design, long-duration spaceflight, reproductive health, space sexology

## Abstract

The imminent transition from low Earth orbit operations to sustained deep-space exploration introduces significant demands upon crew health systems that extend well beyond the physiological and psychological domains traditionally addressed in aerospace medicine. Despite growing recognition that sexuality and reproductive health represent fundamental dimensions of human well-being, these domains have remained conspicuously absent from official mission planning, crew training curricula, and habitat design specifications. This narrative review was therefore undertaken to appraise and synthesize existing evidence on the biological, psychological, ethical, and technological dimensions of human sexuality relevant to long-duration spaceflight and to identify critical knowledge gaps and operational vulnerabilities. A structured literature search was performed across multiple academic repositories, including PubMed, PsycINFO, Web of Science, and Google Scholar, targeting English-language studies published from 1990 to 2026. The scope of the review comprised reproductive physiology within microgravity and high-radiation environments; the psychosocial and psychosexual dynamics of isolated, confined, and extreme settings; the intersection of habitat design, privacy, and ethical-regulatory frameworks; and the emergence of erotic technologies with potential aerospace applications. Eligible studies were thematically analyzed to construct a five-pillar operational framework.

The narrative synthesis identified five operational pillars that require systematic integration into mission architecture. Reproductive risk assessment emerged as a foundational concern, encompassing radiation- and microgravity-induced impairment of gonadal function, gametogenesis, and embryonic development; however, the evidence base remains predominantly derived from animal models and in vitro studies. Psychosexual crew preparedness was identified as equally critical, necessitating the development of consent training, healthy intimacy education, and relationship management strategies. The review further highlighted the need for habitat design standards that incorporate spatial, acoustic, and hygiene requirements for intimate behavior in reduced-gravity environments. Regulatory and ethical governance represents an additional imperative, requiring the establishment of relationship policies, pregnancy contingency protocols, and sexual misconduct reporting mechanisms adapted to the jurisdictional complexities of extraterrestrial operations. Finally, a prioritized research agenda is proposed, targeting systematic reproductive health surveillance, analog-based psychosexual studies, stakeholder consultation, and erobotic technology evaluation. We conclude that the systematic neglect of sexuality and reproductive health in current mission planning may compromise crew well-being, interpersonal dynamics, and ultimately mission success as the duration and remoteness of human spaceflight increase. The proposed framework offers a structured conceptual roadmap for translating space sexology from academic discourse into actionable components of mission architecture but requires empirical validation through analog-based pilot studies and stakeholder engagement. This integration should be prioritized in parallel with the technological development of deep-space transportation systems.

## Introduction and background

The global space enterprise is undergoing a fundamental paradigm shift from established low Earth orbit operations toward deep-space architectures targeting the Moon and Mars [1,2]. NASA's (National Aeronautics and Space Administration's) Artemis program, ESA's (European Space Agency's) contributions to the Lunar Gateway, and commercial ventures led by SpaceX are actively constructing infrastructure for missions lasting months to years, fundamentally redefining the demands placed upon crew health systems [3]. Within this rapidly evolving landscape, however, sexuality and reproductive health remain conspicuously absent from official mission planning documents, crew training curricula, and habitat design specifications - an observation consistently noted in the scholarly literature but not yet empirically quantified through formal institutional analysis [4,5].

The reasons underlying this omission are multifaceted. National and private space organizations have historically avoided sex-related research - a stance attributed by multiple commentators to sociocultural taboos, conservative institutional cultures, and the presumption that short-duration missions rendered such considerations operationally irrelevant [4,6]. Operational perspectives favoring abstinence-based policies, relationship prohibitions, or risk-avoidance strategies may also have contributed to institutional reluctance, alongside concerns related to command authority, liability exposure, and public perception. However, this position has become increasingly untenable as the sector transitions toward multiyear expeditions in which small, mixed-gender crews operate under conditions of extreme isolation and confinement, with Earth-to-crew communication delays extending up to 24 minutes one-way [7,8]. Given that intimacy and sexuality are widely recognized as fundamental human needs with established physiological, psychological, and social dimensions [9,10], their systematic neglect in mission architecture may compromise not only individual crew well-being but also interpersonal dynamics, team cohesion, and, ultimately, mission success [4,11].

The emerging discipline of space sexology - defined as the comprehensive scientific study of human intimacy and sexuality in extraterrestrial environments - was formally articulated by Dubé et al. [4] in a landmark 2023 publication that introduced a biopsychosocial framework for the field. A related scoping review consolidated five years of accumulating research, identifying approximately 50 relevant publications spanning biology, philosophy, ethics, gynecology, aerospace medicine, and sexology [12]. Despite this growing body of scholarship, no operational framework has been proposed to translate space sexology from academic discourse into actionable mission planning tools.

To address this gap, we conducted a narrative review with three principal objectives: (a) to synthesize available evidence across the biological, psychological, ethical, and technological dimensions of human sexuality relevant to long-duration spaceflight; (b) to identify critical knowledge gaps and operational vulnerabilities; and (c) to propose a conceptual operational roadmap organized around five strategic pillars, each mapped onto the sequential phases of mission architecture (pre-mission training, in-flight operations, and post-mission reintegration). The breadth of this integrative approach necessarily limits the depth achievable within each domain. However, we contend that the interdependence of these domains - including reproductive biology, psychosexual health, habitat design, governance, and technology - requires an integrative framework that domain-specific analyses alone cannot provide.

## Review

Methods

Search Strategy

A narrative review was conducted through searches of PubMed, PsycINFO, Web of Science, and Google Scholar electronic databases. Representative Boolean search strings employed in PubMed included the following: (astronaut* OR cosmonaut* OR "space crew") AND (sexual health OR sexuality OR intimacy OR reproduction OR fertility) AND (spaceflight OR microgravity OR "long-duration mission*"); and (isolated confined extreme OR ICE OR confinement) AND (psychosexual OR "romantic relationship*" OR "sexual behavior*"). Equivalent search strategies, adapted to database-specific syntax, were applied across all repositories. Additional sources were consulted, including regulatory databases from NASA [13], the ESA [14], and the European Union Aviation Safety Agency [15], as well as NASA Technical Reports and proceedings from the International Astronautical Congress. Consistent with narrative review methodology, the search strategy was iteratively refined as thematic categories emerged during the review process. The literature review comprised original research articles, reviews, systematic reviews, regulatory documents, and technical reports published in English-language sources between January 1990 and February 2026. The extended time range was necessitated by the limited and historically fragmented nature of literature in this domain.

Eligibility Criteria

Selection criteria specifically targeted studies addressing (a) reproductive physiology in microgravity and radiation environments; (b) psychosocial and psychosexual aspects of isolated, confined, and extreme (ICE) environments; (c) habitat design and privacy in spaceflight contexts; (d) ethical and regulatory frameworks for sexuality and reproduction in space; and (e) erotic technologies with potential space applications (Table 1).

**Table 1:** Search strategy and keywords ICE: Isolated confined extreme; ISS: International Space Station; HI-SEAS: Hawaii space exploration analog and simulation; CHAPEA: Crew health and performance exploration analog; HZE: High, atomic number, and energy; DNA: Deoxyribonucleic acid.

Concept Domain	Search Terms	Variations and Synonyms
Population	Astronaut*	Cosmonaut*, "space crew", "flight crew", "space traveler*"
Setting	Spaceflight	Microgravity, "long-duration mission*", Mars, "deep space", ISS, "space station"
Sexuality/Intimacy	Sexual health	Sexuality, intimacy, "sexual behavior*", psychosexual, eroticism, "romantic relationship*"
Reproduction	Reproduction	Fertility, gametogenesis, embryogenesis, pregnancy, oogenesis, spermatogenesis, contraception
Environment	Isolated confined extreme	ICE, confinement, isolation, "analog environment*", Antarctic, submarine, MARS-500, HI-SEAS, SIRIUS, CHAPEA
Ethics/Policy	Bioethics	"Reproductive rights", consent, governance, policy, "sexual misconduct", "sexual harassment", "sexual assault"
Habitat/Design	Habitability	"Habitat design", privacy, "crew quarters", "space architecture", "personal space"
Technology	Erobotics	"Sex robot*", "erotic technology", telemedicine, "virtual reality", "augmented reality"
Radiation	"Cosmic radiation"	"Space radiation", HZE, "DNA damage", "galactic cosmic rays", "ionizing radiation"

The inclusion of ICE and military analog literature was guided by the established role of these environments as terrestrial proxies for spaceflight conditions in the aerospace medicine literature, sharing critical features including prolonged confinement, isolation, hierarchical command structures, mixed-gender composition, and constrained communication with external support systems. Conference abstracts without full-text availability, non-peer-reviewed preprints, and fictional or speculative narratives without scientific grounding were excluded.

Study Selection Process

**Figure 1:**
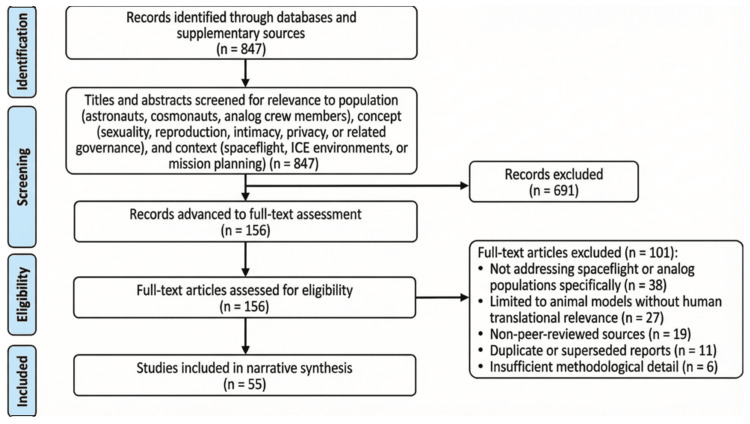
Study selection process

A total of 847 records were initially identified across databases and supplementary sources. Titles and abstracts were screened for relevance to the population (astronauts, cosmonauts, and analog crew members), concept (sexuality, reproduction, intimacy, privacy, or related governance), and context (spaceflight, ICE environments, or mission planning), resulting in 156 records advanced to full-text assessment. Full-text review excluded 101 records for the following reasons: not addressing spaceflight or analog populations specifically (n = 38); limited to animal models without human translational relevance (n = 27); non-peer-reviewed sources (n = 19); duplicate or superseded reports (n = 11); and insufficient methodological detail (n = 6). A final set of 55 studies met the inclusion criteria and were included in the narrative synthesis (Figure 1).

Data Extraction and Thematic Analysis

**Figure 2:**
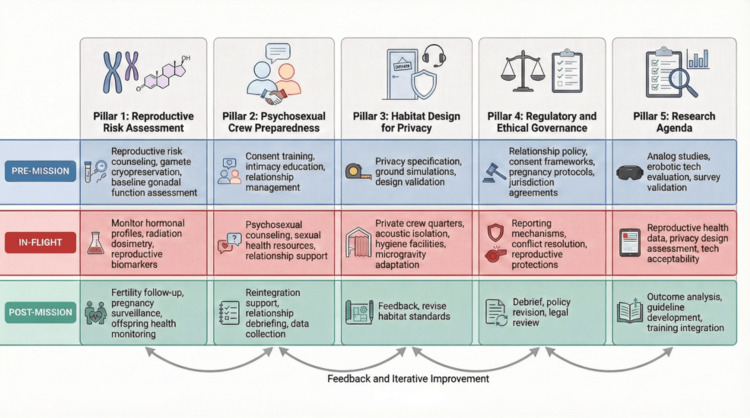
Five-pillar operational framework for space sexology integration across mission phases The proposed framework illustrates the systematic integration of space sexology considerations throughout the complete mission lifecycle, organized into five strategic pillars and three temporal phases. Each pillar addresses a distinct operational domain: (Pillar 1) Reproductive Risk Assessment comprises biological monitoring and fertility protection; (Pillar 2) Psychosexual Crew Preparedness includes training, counseling, and relationship management; (Pillar 3) Habitat Design for Privacy addresses spatial, acoustic, and hygiene requirements; (Pillar 4) Regulatory and Ethical Governance establishes policies, protocols, and reporting mechanisms; (Pillar 5) Research Agenda prioritizes knowledge generation across analog studies, technology evaluation, stakeholder consultation, and surveillance systems. The three temporal phases - pre-mission (blue), in-flight (red), and post-mission (green) - define the implementation timeline for each pillar's specific interventions. Arrows indicate information flow and feedback loops between phases, emphasizing the iterative nature of operational learning. The framework is intended as a conceptual architectural blueprint requiring empirical validation through analog-based pilot studies and structured stakeholder engagement prior to operational deployment. Credit: This image was created by the author, Piercarlo Minoretti, using the AI-assisted illustration tool FigureLabs.ai.

The available literature was critically appraised and subsequently thematically analyzed to construct a five-pillar operational framework (Figure 2). Given the extreme methodological heterogeneity of the included literature - spanning preclinical animal studies, observational human research, analog-based behavioral investigations, theoretical and bioethical analyses, regulatory documents, and technical reports - a narrative thematic synthesis was selected over quantitative summary methods such as meta-analysis or meta-regression. Standardized quality appraisal instruments (e.g., Newcastle-Ottawa Scale, ROBINS-I (Risk of Bias in Non-randomized Studies - of Interventions), and GRADE (Grading of Recommendations Assessment, Development, and Evaluation)) were not applied, as these tools are calibrated for specific study architectures (e.g., cohort, case-control, and randomized trials) that represent a minority of the included sources; their application would have either excluded the majority of relevant scholarship or produced misleading quality ratings. This methodological choice is acknowledged as a limitation, and future domain-specific systematic reviews with formal quality grading are warranted.

Results

Pillar 1: Reproductive Risk Assessment

The effects of the space environment on reproductive physiology represent the most extensively studied domain within the broader landscape of space sexology. Accordingly, two primary hazards, such as cosmic radiation and microgravity, have been demonstrated to independently and synergistically impair reproductive function across multiple biological levels [16-18]. It should be noted, however, that the evidence base for these effects remains predominantly derived from animal models and in vitro studies, and the translational validity of these findings to human spaceflight contexts has not been established.

Concerning male reproductive health, exposure to heavy ion radiation and simulated microgravity induces spermatogenic cell apoptosis, DNA fragmentation, and chromatin structural abnormalities in rodent models [19]. A notable International Space Station (ISS) study, however, demonstrated that freeze-dried mouse spermatozoa preserved aboard the ISS for up to six years retained reproductive normality, producing genetically healthy offspring at rates comparable to ground controls [20]. While encouraging, these findings reflect cryopreserved gamete resilience rather than in vivo spermatogenesis under chronic space exposure. Additional rodent studies have documented testosterone synthesis disruption and testicular atrophy following simulated microgravity exposure, though corresponding human data remain absent [16]. In contrast to the mixed evidence for male fertility, female reproductive systems appear particularly vulnerable to spaceflight conditions based on available preclinical data. Murine models have demonstrated cessation of estrous cycling, loss of corpora lutea, and reduced estrogen receptor expression following space exposure [21,22]. The combined effects of radiation on oocyte reserves raise especially serious concerns, as these non-regenerative cells - unlike continuously produced spermatogonia - cannot be replaced following damage [16,18]. Further complicating this landscape, hormonal contraception commonly recommended by flight surgeons for menstrual suppression during missions may interact with spaceflight-associated neuroendocrine changes in ways that remain poorly characterized [21].

Beyond gamete integrity, embryonic development in microgravity presents additional challenges. Early mammalian development following fertilization demonstrates increased frequencies of abnormal cell division and developmental arrest under both simulated and actual microgravity conditions [23,24]. While fertilization itself appears not to be gravity-dependent in vitro [23], the post-fertilization developmental cascade exhibits significant sensitivity to gravitational perturbation, with potentially significant implications for any future scenarios involving conception in space [25,26]. Recognizing the complexity and multifaceted nature of these reproductive challenges, two recently published comprehensive reviews have substantially advanced the field's theoretical foundations and practical frameworks. Accordingly, a systematic review highlighted the mechanistic roles of extracellular vesicles, microbiome shifts, and oxidative stress in microgravity-related reproductive impairment while discussing potential countermeasures including artificial gravity and antioxidant strategies [17]. Complementing this mechanistic perspective, a bioethics-focused analysis addressed the ethical dimensions of reproductive biomedicine in the era of commercial spaceflight, noting the increasing urgency as non-professional astronauts of all ages participate in space missions [27]. An additional technical assessment examined the feasibility of adapting assisted reproductive technologies for space environments, identifying critical challenges in gamete handling, embryo culture, and cryopreservation under non-terrestrial conditions [28].

Despite this substantial and growing body of evidence, systematic reproductive health monitoring of astronauts before, during, and after missions remains severely limited. In this regard, reproductive outcomes following spaceflight - including fertility rates, pregnancy complications, and congenital anomalies - have not been systematically reported for either male or female astronauts [21,29]. This surveillance gap represents a significant operational and ethical vulnerability as mission durations extend and crew demographics diversify and constitutes one of the most pressing justifications for implementing the longitudinal monitoring protocols proposed in the research agenda.

Pillar 2: Psychosexual Crew Preparedness

The psychological and interpersonal challenges of ICE environments have been extensively studied, providing a robust - though incomplete - foundation for understanding the psychosexual dimensions of long-duration spaceflight [30-32]. A comprehensive review of psychosocial issues across individual, interpersonal, and organizational domains documented phenomena including emotional lability, cognitive performance fluctuations, crew tension and conflict, subgroup formation, and the critical importance of leadership and coping strategies [30]. Building upon these theoretical foundations, the 520-day Mars-500 simulation provided empirical evidence of substantial interindividual variability in behavioral responses to prolonged confinement, with notable decreases in activity levels, degradation in sleep quality, and increases in psychological distress - particularly during the mission's middle phase [33]. Layered onto these individual-level challenges, cross-cultural dynamics introduce additional complexity to crew interpersonal functioning. Results from the SFINCSS-99 (Simulation of Flight of International Crew on Space Station) study demonstrated that cultural and language differences can hinder the formation of joint cohesive crews, with subjects perceiving intergroup members as increasingly distant over time [34]. Perhaps most relevant to understanding paired crew dynamics, a unique study of a three-couple Arctic expedition - representing the closest analog to mixed-gender partnered crews - found that couples demonstrated higher positive than negative affect overall, although negative events peaked at specific mission phases [35]. In response to these documented interpersonal challenges, behavioral health countermeasures tested at the Hawaii Space Exploration Analog and Simulation (HI-SEAS) Mars analog, including computer-based conflict resolution and stress management programs, showed promise but were not designed to address intimate or sexual dimensions of crew interaction [36,37].

Despite this growing body of ICE environment research, the critical gap in this domain is the complete absence of psychosexual preparedness protocols. No published study has examined consent training, healthy intimacy education, relationship management strategies, or conflict resolution specifically addressing romantic or sexual interactions among crew members. It is worth noting that confined occupational environments with longer operational histories - including submarines, Antarctic research stations, and offshore platforms - have accumulated substantial observational data on interpersonal dynamics and intimate relationships under confinement. A dedicated cross-occupational comparative analysis of these settings would provide a stronger translational foundation for spaceflight-specific recommendations, although such an analysis was beyond the scope of the present review. Relevant insights may also be drawn from the military literature on sexual harassment and assault prevention in confined environments, which may offer a partial template. Accordingly, research in this domain has documented the unique interplay between harassment and assault in military contexts, emphasizing how confined settings, power hierarchies, and limited reporting options compound risk [38]. A synthesis of evaluations of military sexual assault prevention programs revealed that most relied on awareness-building rather than evidence-based skill development [39]. These military analogs highlight both the urgency and the methodological foundations that could inform the development of spaceflight-specific psychosexual training curricula.

Balancing risk mitigation with positive outcomes, the potential advantages of enabling - rather than suppressing - intimacy in space merit careful consideration. Sexual activity is associated with physiological benefits including stress reduction, pain alleviation, improved sleep quality, and immune modulation [4,12]. In the context of prolonged isolation, these benefits may serve as adaptive countermeasures. However, the risks of interpersonal conflict, favoritism, power imbalances, and post-relationship tension within small crews are equally significant and must be proactively managed [4,11]. It should be acknowledged that operational perspectives favoring abstinence policies or strict relationship prohibitions during missions represent a legitimate risk-management position, particularly given concerns related to command authority, crew dynamics, and mission integrity. The present review does not dismiss these perspectives but contends that their long-term viability diminishes as mission durations extend into multiyear timeframes and crew autonomy increases proportionally to Earth-communication delays.

Pillar 3: Habitat Design for Privacy

While the architecture of space habitats profoundly influences crew psychological well-being, current designs provide minimal accommodation for intimate privacy [40,41]. On the ISS, crew quarters measure approximately 2.1 cubic meters, which are sufficient for sleeping and basic personal activities but inadequate for the privacy requirements of long-duration missions [42]. Recognizing these spatial constraints, NASA's Deep Space Habitability Design Guidelines, derived from the NextSTEP Phase 2 ground test program, identified private crew quarters and dedicated hygiene compartments as essential capabilities for missions exceeding 30 days [42]. Critically, however, these guidelines address privacy exclusively in terms of sleep, personal communication, and hygiene, with no reference to intimate or sexual privacy needs. To fully understand these deficits, the environmental psychology of capsule habitats has established that privacy functions along multiple dimensions (i.e., visual, acoustic, informational, and territorial) [43], which become acutely relevant when considering intimate behavior in confined environments. Acoustic isolation is particularly challenging in pressurized habitats where structural vibration transmission and ventilation noise create environments of near-continuous auditory permeability [40]. Compounding these limitations, the absence of transitional and buffer zones between private and communal areas has been identified as a significant source of stress in analog facilities, producing perceptions of intrusion and surveillance [40,44]. Addressing these multifaceted privacy challenges, design solutions from the emerging field of space architecture offer potential approaches. Potential innovations include adaptive partition systems capable of reconfiguring spaces for varying privacy levels, personal acoustic environments employing active noise cancellation, and the integration of biophilic design elements to create psychologically restorative private spaces [40,44]. Despite these architectural advances, however, no published habitat design has specifically addressed the spatial, acoustic, and hygiene requirements for sexual activity in reduced-gravity or microgravity environments. The empirical validation of privacy-enabling design solutions remains a necessary prerequisite for their incorporation into flight-ready habitat modules, and ground-based simulation testing using partial-gravity analogs should be prioritized to evaluate proposed configurations before orbital implementation.

Pillar 4: Regulatory and Ethical Governance

The absence of formal governance frameworks addressing sexuality in space represents perhaps the most consequential gap identified in the present review. Current regulatory structures from NASA, ESA, and other agencies contain no published policies regarding consensual sexual relationships among crew members, pregnancy contingency planning, sexual misconduct reporting mechanisms adapted for deep-space communication delays, or reproductive health monitoring protocols [4,12,45]. It should be acknowledged that this regulatory silence may not be entirely attributable to institutional oversight. Operational perspectives favoring risk-avoidance strategies, concerns regarding command authority and liability exposure, and sensitivities related to public perception may all represent rational - if ultimately insufficient - factors contributing to institutional reluctance to formalize governance in this domain.

In response to this regulatory vacuum, the bioethics of space reproduction has received growing scholarly attention. An initial examination of whether space bioethics raises genuinely novel challenges concluded that while the issues parallel those encountered in military ethics, extreme environments, and settings of curtailed autonomy, the combination of existential risk and unprecedented isolation creates uniquely weighted ethical considerations [45]. Expanding upon this foundation, subsequent work argued for a feminist space bioethics that foregrounds reproductive autonomy and gender equity in mission planning and governance [46]. These theoretical frameworks have been further developed through analyses of the biological and social challenges of human reproduction in a long-term Mars base, which highlighted the tension between collective survival imperatives and individual reproductive rights in scenarios where population genetics, resource constraints, and environmental hazards may necessitate reproductive regulation [47]. While these contributions provide a valuable theoretical foundation, the governance and ethical analysis in this domain remain predominantly at the level of scholarly commentary. Detailed legal examination - encompassing the Outer Space Treaty, bilateral intergovernmental agreements governing ISS operations, national employment and military law, and emerging commercial spaceflight regulations - has not been systematically undertaken and will require dedicated collaboration with space law specialists and comparative jurisprudence experts.

Drawing lessons from terrestrial analogs, parallels from military contexts are instructive. The US Department of Defense has invested substantial resources in sexual assault and harassment prevention; however, prevalence rates remain persistently high despite formal policies and training programs [38,39,48]. This experience suggests that governance frameworks alone are insufficient without accompanying cultural change, confidential reporting mechanisms, accessible support services, and meaningful accountability structures. In the spaceflight context, these challenges are compounded by the impossibility of physical separation between involved parties, communication delays precluding real-time ground-based intervention, and the absence of legal jurisdictional clarity in extraterrestrial environments.

Among the various governance challenges, pregnancy in space presents a particularly complex scenario. NASA medical standards disqualify pregnant flight crew members [49]. However, for multiyear missions, contraceptive failure, changes in reproductive intent, and the theoretical possibility of conception in the absence of strict prevention measures necessitate contingency protocols. A critical and unresolved policy question is whether conception during exploration-class missions should be treated as an anticipated operational scenario requiring accommodation or as a preventable risk to be actively mitigated through strict countermeasures. The latter position carries considerable weight given the serious ethical concerns surrounding conception in space, including medical uncertainty regarding fetal development in radiation and microgravity environments, the inability to obtain meaningful informed consent for the future child, and potential compromises to mission safety and crew welfare. The present review does not advocate for the normalization of pregnancy during exploration-class missions; rather, it contends that contingency planning remains essential regardless of which policy stance is adopted, because even with rigorous prevention measures, contraceptive failure rates and changes in reproductive intent cannot be eliminated over multiyear mission timescales. These contingency protocols must address medical management of pregnancy in microgravity and radiation environments, mission-impact assessment, crew role redistribution, and ethical frameworks for decision-making that respect reproductive autonomy while acknowledging collective mission imperatives [27,45].

Pillar 5: Prioritized Research Agenda

Translating space sexology from advocacy to operational capability requires a structured research agenda. Based on the available literature, six priority domains are identified.

First, systematic reproductive health surveillance of astronauts should be implemented, including longitudinal monitoring of gonadal function, gamete quality, hormonal profiles, and reproductive outcomes. The NASA Twins Study demonstrated the feasibility and value of comprehensive multi-omic monitoring during long-duration spaceflight [3]; similar approaches should be extended to reproductive endpoints.

Second, building upon this biological surveillance foundation, analog-based studies should formally incorporate psychosexual variables into their research protocols. While the Mars-500, SIRIUS, HI-SEAS, and CHAPEA programs have generated valuable data on crew psychology and interpersonal dynamics [33,36,37,50], none have systematically studied sexual behavior, intimate relationship dynamics, or the effects of sexual abstinence on crew well-being. Given the sensitivity of this topic, validated anonymous survey instruments and opt-in qualitative protocols should be developed.

Third, complementing these human-factor investigations, habitat design research should explicitly address privacy requirements for intimate behavior, encompassing spatial configurations, acoustic isolation standards, hygiene protocols, and microgravity-adapted design solutions. Ground-based simulations using partial-gravity analogs could evaluate proposed designs prior to orbital implementation.

Fourth, alongside these architectural solutions, the emerging field of erobotics - defined as the transdisciplinary study of human-machine erotic interaction - offers potential technological solutions for space applications [51,52]. Beyond their role as adjuncts to human intimacy, erobotic systems - including immersive virtual reality platforms, augmented reality interfaces, and tele-intimacy technologies - merit evaluation as structured harm-reduction strategies capable of addressing psychosocial and sexual needs while mitigating the interpersonal risks inherent in human-human sexual relationships within small, high-risk crews. Specifically, technology-mediated intimacy may reduce exposure to power asymmetries, pregnancy risk, favoritism dynamics, and post-relationship crew disruption that represent significant operational concerns when sexual relationships develop between crew members. A comparative assessment of the relative risk profiles, ethical implications, engineering feasibility, and psychological consequences of human-human versus human-technology intimacy should be considered a high-priority research objective. Tele-intimacy technologies may additionally serve telemedicine functions for maintaining intimate connections with Earth-based partners, addressing the social and emotional dimensions of prolonged separation. It should be acknowledged, however, that no aerospace-specific feasibility data currently exist for any of these technologies; critical technical challenges - including mass and volume constraints, power limitations, hygiene requirements in microgravity, and the psychological effects of prolonged reliance on technology-mediated intimacy - remain entirely uncharacterized and require dedicated investigation in analog environments prior to any operational deployment. To ensure responsible implementation, research should evaluate the acceptability, efficacy, and psychological effects of such technologies in analog environments.

Integrating these technical and scientific advances within an appropriate regulatory structure, fifth, governance frameworks should be developed through interdisciplinary collaboration among aerospace medicine specialists, bioethicists, legal scholars, psychologists, and astronaut representatives. These frameworks should address relationship policies, consent protocols adapted for hierarchical and confined settings, pregnancy contingency planning, confidential reporting mechanisms for sexual misconduct, and reproductive health protections.

Sixth, structured stakeholder consultation should be undertaken as a necessary validation step prior to operational implementation of any component of the proposed framework. Direct input from astronauts, flight surgeons, behavioral health specialists, habitat engineers, and policymakers - obtained through methods such as Delphi surveys, expert panels, and astronaut focus groups - is essential for translating the present literature-based framework from conceptual architecture into implementable protocols with ecological validity and practical applicability. The absence of such stakeholder input in the present review is acknowledged as a limitation.

Discussion

The present narrative review revealed a fundamental paradox in current approaches to crew health in long-duration spaceflight. While virtually every other dimension of human physiology and psychology has been subject to intensive study and countermeasure development, sexuality and reproductive health remain systematically neglected [4,12]. Critically, this neglect is not attributable to a lack of scientific importance but rather to institutional reluctance that has been attributed by multiple commentators to sociocultural taboos and the historical focus on short-duration missions where such considerations could be deferred [6]. It should be recognized, however, that institutional caution may also reflect legitimate operational considerations - including command-structure concerns, liability exposure, public-perception sensitivities, and the pragmatic judgment that abstinence-based or restrictive policies represent a simpler risk-management approach. While these perspectives have operational logic, particularly for shorter missions, their long-term sustainability is questionable as mission durations extend into multiyear timeframes and crew autonomy increases proportionally to Earth-communication delays.

Without timely intervention, the consequences of continued inaction are potentially severe and extend across multiple operational domains. At the biological level, uncharacterized reproductive risks may compromise the long-term health of astronauts and, in scenarios involving conception, the viability of offspring [16-18], though it must be emphasized that these risks remain predominantly extrapolated from animal models and in vitro studies, and their direct applicability to human spaceflight has not been confirmed. Compounding these physiological concerns, at the psychological level, the suppression of basic human needs for intimacy and sexual expression during multiyear missions may exacerbate the already substantial risks of behavioral health decrements, interpersonal conflict, and psychological deterioration [4,30,53]. Beyond individual and interpersonal impacts, at the organizational level, the absence of governance frameworks creates vulnerability to sexual misconduct, relationship-driven crew dysfunction, and legal and ethical controversies that could undermine public support for space programs [38,45].

To systematically address these multifaceted vulnerabilities, the five-pillar operational framework proposed in this review offers a structured conceptual approach spanning the sequential phases of mission architecture (Figure 2).

Specifically, pre-mission interventions should comprise reproductive risk counseling (including gamete cryopreservation options), psychosexual preparedness training, and establishment of governance expectations. In-flight support should comprise privacy-enabling habitat design, accessible and confidential sexual health resources, and mechanisms for relationship management and conflict resolution. Completing the mission lifecycle, post-mission protocols should address reproductive health monitoring, psychological reintegration support for relationship changes that may have occurred during missions, and systematic debriefing to inform future mission planning.

The operational salience of the proposed framework increases as a function of several mission parameters, including duration, crew size, Earth-communication delay, and degree of crew autonomy. Future governance frameworks should consider these parameters when calibrating the scope and stringency of sexuality-related policies and interventions rather than applying uniform protocols across mission profiles of fundamentally different character.

A critical and unresolved policy question concerns the operational posture toward pregnancy during exploration-class missions. The distinction between a prevention-first approach, in which conception is treated as a risk to be actively mitigated through strict countermeasures, and an accommodation approach, in which pregnancy is treated as an anticipated scenario requiring contingency planning, carries profound ethical, medical, and organizational implications. The serious concerns surrounding conception in space, including medical uncertainty regarding fetal development under radiation and microgravity exposure, the inability to obtain meaningful informed consent for the future child, and potential compromises to mission safety, lend considerable weight to a prevention-first posture. The present review does not advocate for the normalization of pregnancy during exploration-class missions. It does, however, maintain that contingency protocols remain essential regardless of which policy stance is adopted, as contraceptive failure rates and changes in reproductive intent cannot be eliminated over multiyear mission timescales.

The potential role of erobotic and tele-intimacy technologies merits particular attention within this framework. Rather than functioning solely as speculative adjuncts, these technologies may represent operationally controllable alternatives to interpersonal sexual relationships, offering a structured harm-reduction approach that addresses psychosocial and sexual needs while mitigating the risks of power asymmetry, pregnancy, favoritism, and post-relationship crew disruption. A comparative evaluation of human-human versus human-technology intimacy across these dimensions should be prioritized. At present, however, no aerospace-specific feasibility data exist, and the technical challenges of deploying such systems in space environments - including mass and volume constraints, power limitations, hygiene requirements, and the psychological effects of prolonged reliance on technology-mediated intimacy - remain entirely uncharacterized.

The value of comparative occupational analysis should also be acknowledged. Confined operational environments with longer institutional histories - including submarines, Antarctic research stations, offshore platforms, and military deployments - have accumulated decades of observational data on interpersonal dynamics, intimate relationships, and sexual behavior under confinement. A systematic cross-occupational analysis of these settings would provide a stronger translational foundation for spaceflight-specific recommendations than the currently available literature permits and should be considered a high-priority avenue for future investigation.

Translating this framework into operational practice, the integration of space sexology into aerospace medicine will require overcoming several barriers. First, institutional resistance must be addressed through leadership engagement and the normalization of sexuality as a legitimate component of crew health, paralleling ongoing efforts to destigmatize mental health in aviation [54,55]. Second, research funding must be allocated specifically to the domains identified in the research agenda, with particular emphasis on analog-based studies capable of generating actionable data without the constraints and costs of orbital research. Third and finally, international coordination is necessary, as crews will increasingly be multinational and governed by heterogeneous regulatory frameworks reflecting diverse cultural attitudes toward sexuality [34,45]. Among the five pillars, reproductive risk assessment and regulatory governance should be considered the most time-sensitive priorities. Reproductive risk counseling and gamete cryopreservation protocols can be implemented within existing pre-flight medical frameworks with relatively modest investment, whereas governance structures require protracted multilateral negotiation and should therefore be initiated well in advance of mission timelines. By contrast, habitat redesign and erobotic technology evaluation represent long-term objectives that can be addressed iteratively, as deep-space habitation modules progress through design phases.

Several limitations of the present review should be acknowledged. The narrative design, while appropriate for synthesizing a fragmented and interdisciplinary literature, does not permit quantitative meta-analysis and carries an inherent degree of subjectivity in study selection and thematic synthesis. No standardized risk-of-bias assessment, quality grading framework, or inter-rater reliability analysis was applied, as the extreme methodological heterogeneity of the included literature precluded the use of tools calibrated for homogeneous study architectures; this methodological choice limits the capacity to quantitatively establish the evidentiary strength of the proposed five-pillar framework. The review does not meet PRISMA-level reporting standards, which were not the methodological target; however, several PRISMA-aligned elements - including a flow diagram, structured search table, and explicit exclusion criteria - have been incorporated to enhance transparency. In addition, the available literature is heavily weighted toward animal models and theoretical frameworks, with limited human data from actual spaceflight. Consequently, several conclusions regarding fertility impairment, embryogenesis risk, and hormonal disruption are necessarily extrapolative, and their translational validity to human spaceflight contexts remains unconfirmed. Furthermore, publication bias likely underestimates the extent of research conducted but not published due to institutional sensitivities. The governance and ethical analysis, while theoretically informed, does not include detailed legal examination of the overlapping jurisdictional frameworks - such as the Outer Space Treaty, bilateral intergovernmental agreements, national employment law, and commercial spaceflight regulations - that will govern the practical implementation of the proposed recommendations; interdisciplinary collaboration with space law specialists is essential for this purpose. Finally, analog-based findings may not fully translate to the unique stressors of actual deep-space missions. The operational framework remains at the conceptual stage and has not been empirically validated through pilot implementation studies, feasibility trials, cost analyses, or engineering integration assessments. Phased validation through analog-based pilot studies, stakeholder consultation, and iterative testing is a necessary prerequisite for operational deployment. In this regard, the framework has not incorporated direct stakeholder input from astronauts, flight surgeons, behavioral health specialists, habitat engineers, or policymakers. Structured stakeholder consultation - through methods such as Delphi surveys, expert panels, and astronaut focus groups - represents a critical next step that should precede any pilot implementation. Each pillar of the proposed framework warrants dedicated, in-depth investigation through domain-specific systematic reviews, and the present work is intended as an integrative architectural blueprint rather than an exhaustive treatment of any single domain.

## Conclusions

Space sexology must transition from academic advocacy to operational implementation. The five-pillar framework proposed in the present review - comprising reproductive risk assessment, psychosexual crew preparedness, habitat design for privacy, regulatory and ethical governance, and a prioritized research agenda - provides a structured conceptual roadmap for this transition. The framework remains at the pre-empirical stage and requires phased validation through analog-based pilot studies, engineering integration assessments, stakeholder consultation, and iterative testing before operational deployment can be considered. Building upon this foundation, we propose that aerospace medicine organizations, including NASA, ESA, and their international partners, formally integrate sexuality and reproductive health into their human research programs and mission planning processes. To operationalize this integration, the development of evidence-based guidelines, training protocols, habitat standards, and governance frameworks should be prioritized in parallel with the technological development of deep-space transportation systems. This process should be informed by direct input from operational stakeholders - including astronauts, flight surgeons, behavioral health specialists, habitat engineers, and policymakers - and should draw upon comparative occupational data from confined environments with longer institutional histories, including submarines, Antarctic research stations, and offshore platforms.

International and interdisciplinary coordination will be essential, as the governance, legal, and ethical dimensions of this domain require collaboration among aerospace medicine specialists, bioethicists, space law experts, psychologists, and astronaut representatives. The stakes are considerable: continued neglect of these fundamental human needs risks compromising crew well-being, interpersonal dynamics, and ultimately mission success. The operational relevance of the proposed framework increases as a function of mission duration, crew size, Earth-communication delay, and degree of crew autonomy. Accordingly, the scope and stringency of sexuality-related policies and interventions should be calibrated to specific mission profiles rather than applied uniformly. With crewed Mars missions now projected within the 2030s-2040s timeframe, the window for developing, testing, and validating these systems in analog and lunar environments is narrowing. The imperative is no longer whether to integrate space sexology into mission architecture but how rapidly this integration can be achieved.
